# Fetal cardiac MRI: a single center experience over 14-years on the potential utility as an adjunct to fetal technically inadequate echocardiography

**DOI:** 10.1038/s41598-020-69375-3

**Published:** 2020-07-23

**Authors:** Su-Zhen Dong, Ming Zhu, Hui Ji, Jing-Ya Ren, Ke Liu

**Affiliations:** Department of Radiology, Shanghai Children’s Medical Center, Shanghai Jiaotong University School of Medicine, Shanghai, 200127 China

**Keywords:** Magnetic resonance imaging, Congenital heart defects

## Abstract

Unlike ultrasound (US) imaging, foetal magnetic resonance imaging (MRI) is not significantly limited by maternal obesity, oligohydramnios, uterine myoma, twins, and foetal lie, which impair US visualization of the foetus. The present study aimed to introduce our foetal cardiac MRI scanning technology and over 14-years of experience on the potential utility of foetal cardiac MRI examination as an adjunct to foetal technically inadequate echocardiography (Echo). This retrospective review included 1,573 pregnant women [1,619 foetuses (46 twins)] referred for a foetal cardiac MRI because of technically limited Echo. Foetal cardiac MRI was performed using two 1.5 T units. Among the 1,619 foetuses referred for cardiac MRI, 1,379 (85.2%) cases were followed up using postnatal imaging and/or surgery, 240 (14.8%), including three twins, had no follow-up confirmation because of pregnancy termination without autopsy or loss to follow-up. The results of the present study indicated that foetal cardiac MRI examinations can be a useful adjunct to foetal echocardiography when the technical limitations of echocardiography make it inadequate for diagnosis.

## Introduction

The foetal cardiovascular system is the most frequently affected among congenital pathologies. Congenital heart disease (CHD) is the most common congenital anomaly and is the major cause of infant mortality^1^. Prenatal diagnosis of certain types of CHD, such as transposition of the great arteries (TGA), pulmonary atresia with intact ventricle septum (PA/IVS), hypoplastic left heart syndrome (HLHS), obstructive total anomalous pulmonary veins connection (TAPVC), and other severe CHDs has been associated with improved outcomes and decreased perioperative mortality^2^. Foetal cardiac examination has become a routine part of screening foetal ultrasound (US), and suspected cardiac anomalies will require more comprehensive evaluation using foetal echocardiography (Echo). Foetal magnetic resonance imaging (MRI) examinations have become useful adjuncts to ultrasound (US) exams when US diagnosis is doubtful. To date, there has been no clinical or experimental evidence that MRI has any adverse effects on the human foetus in the second or third trimester^3,4^. Unlike US imaging, foetal MRI is not significantly limited by maternal obesity, oligohydramnios, uterine myoma, twins, and foetal lie, all of which impair US visualization of the foetus^5–7^.

The visualization of the foetal heart using MRI is complicated because of the small heart size, high heart rate, and foetal motion^4,8^. Several studies revealed new techniques (such as compressed sensing, motion correction, and foetal cardiac gating) to reveal further details in MRI studies of the foetal heart and great vessels^10–13^. Foetal cardiac MRI has the potential to complement echocardiography to detect cardiovascular anomalies^14,15^. However, currently, there has been no report on the potential utility of foetal cardiovascular MRI as an adjunct to foetal echocardiography when echocardiography is technically limited, either from a single centre or as pooled data from multiple centres. In this study, we introduce our special foetal cardiovascular MRI scanning techniques and experience of the potential utility of foetal cardiovascular MRI as an adjunct to technically inadequate foetal echocardiography.

## Methods

### Subjects

The ethics committee of Shanghai Children’s Medical Center approved the study. Written informed consent and permission for imaging were obtained from all pregnant mothers before MRI. All methods were performed in accordance with the relevant guidelines and regulations. This retrospective review included 1,573 pregnant women [1,619 foetuses (46 twins)] referred to a children’s hospital for a foetal cardiac MRI from January 2005 to October 2019 as a result of technically limited Echo. Limitations of foetal echocardiography were attributed to maternal obesity (body mass index ≥ 30 kg/m^2^), maternal abdominal wall oedema, uterine myomas, oligohydramnios [maximum vertical pocket depth < 2 cm or four quadrants amniotic fluid index (AFI) < 5 cm], twins (n = 46), and unfavourable foetal lie (Table 1)^5,16,17^. In 1,573 pregnant women, 46 pregnant women carried twins (including three pairs of conjoined twins); therefore, the study included a total of 1,619 foetuses. The study only included selected cases in which the foetal echocardiography findings were inadequate to show the four chambers of the heart and/or the outflow tracts of the two ventricles because of the technical limitations of repeated Echo. Colleagues who interpreted the MRI data were blinded to the outcomes of the foetal echocardiography examinations. Follow-up diagnosis was confirmed using postnatal imaging or surgery.

**Table 1:** Limitations for fetal echocardiography.

Limitations for fetal echocardiography	No. of fetuses
Maternal obesity	1,082
Maternal abdominal wall edema	42
Uterine myomas	21
Oligohydramnios	70
Twins and unfavorable fetal position	92 (46 twins)
Unfavorable fetal lie	312

In this group of 1,573 pregnant women, 1,249 (79.4%) cases had risk factors for pregnancy, including maternal age over 35, maternal obesity, maternal diabetes mellitus, maternal hypertension, multiple pregnancies, IVF baby, a history of a previous pregnancy with CHD, and a family history of CHD.

### MRI acquisition

Foetal cardiac MRI was performed at two 1.5 T units (Signa Echospeed; GE Medical Systems, Milwaukee, WI, USA; and Achieva Nova dual; Philips Medical Systems, Best, The Netherlands). 1,069 pregnant women were assessed using the Philips 1.5 T unit and 504 pregnant women were assessed using the GE 1.5 T unit. Between January 2005 and December 2010, foetal cardiac MRI was performed using the GE 1.5-T unit (33 mT/m gradients and an eight-channel phased array cardiac coil). Between December 2010 and October 2019, foetal cardiac MRI was performed using the Philips 1.5-T unit (60 mT/m gradients and a sixteen-channel sense-xl-torso coil). Gestational age ranged from 22 to 36 weeks (mean 24.5 weeks). The age of the pregnant women ranged from 20 to 42 years (mean 29.5 years).

**Figure 1:**
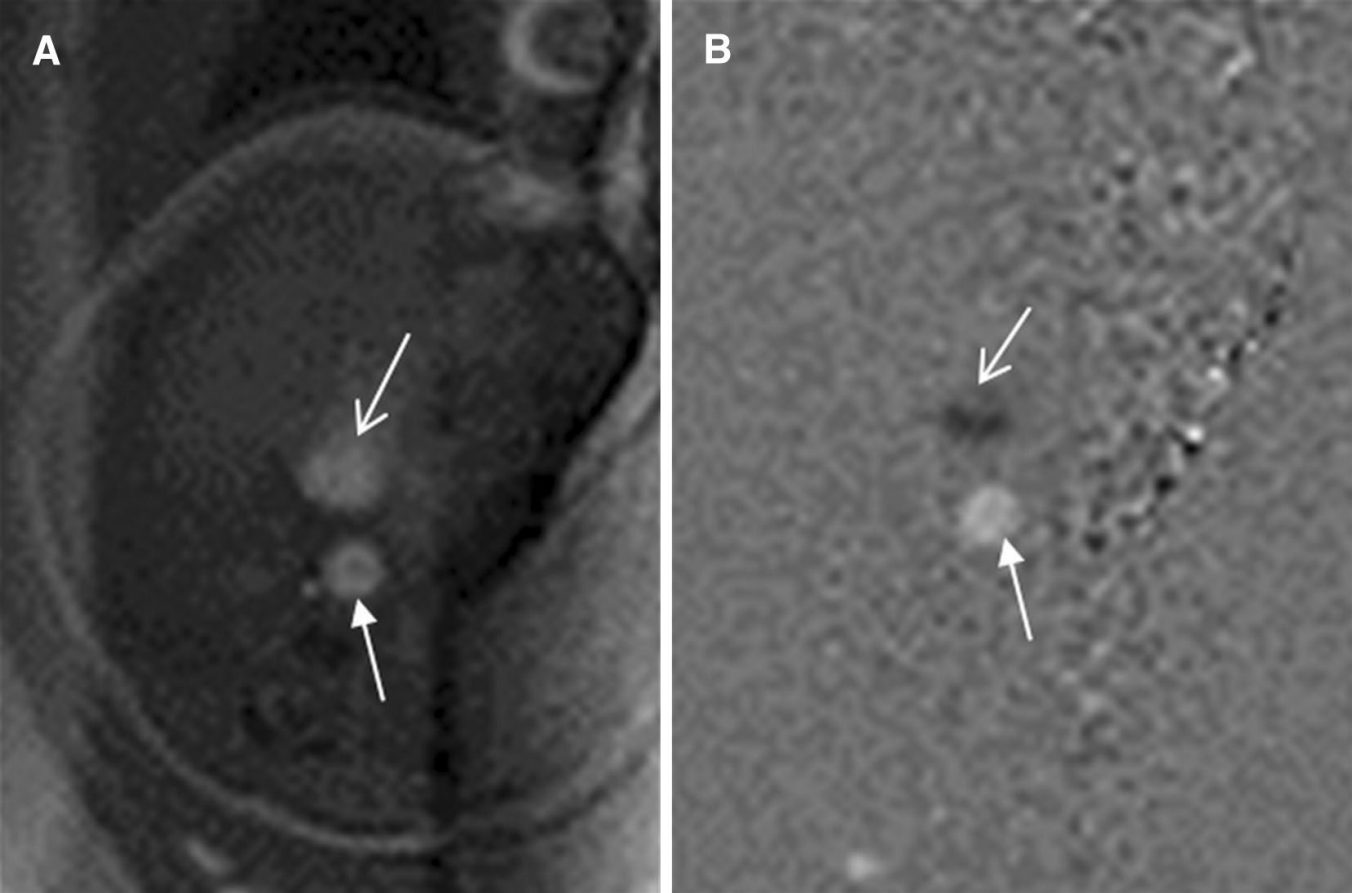
A 29-week-old foetus with a non-gated phase-contrast sequence. Foetal CMR non-gated phase-contrast sequence showing that the flow directions of the inferior vena cava (open arrows in **A**,**B**; black in **B**) and descending aorta are opposite (arrows in **A**,**B**; white in **B**).

Imaging sequences included steady-state free-precession (SSFP), real-time SSFP, single-shot turbo spin echo (SSTSE), and non-gated phase contrast (PC) sequences. The SSFP sequence was the most important sequence because of its better contrast between blood flow (hyper-intensity) and myocardium (hypo-intensity). An SSTSE sequence [Repetition Time (TR)/Echo Time (TE), 12,000/120 ms; field of view (FOV), 280–335 mm^2^; section thickness, 4–6 mm; spacing, 0–0.5 mm; matrix, 192 × 193–300 × 222; flip angle, 90°] was acquired mainly in an oblique coronal and axial view to assess the bronchus and visceroatrial situs. An SSFP sequence (TR/TE, 3.6/1.8 ms; FOV, 260–325 mm^2^; section thickness, 4–6 mm; overlapping, 3–5 mm; matrix, 192 × 193–232 × 230; flip angle, 70°–80°) was mostly acquired in the transverse view of the foetal thorax; the four-chamber plane; the short-axis, coronal, and oblique sagittal planes of the foetal heart; and especially, the transverse view of the aortic arch and the four chambers were most important planes. A real-time SSFP sequence was acquired along the transverse and short-axis planes of the foetal heart to show the beating heart. A real-time PC sequence without cardiac gating was used to show the blood flow direction (Fig. 1; Table 2).

**Table 2:** All scanning parameters of fetal CMR sequences.

	TR (ms)	TE (ms)	FOV (mm)	Matrix	Slice thickness (mm)	Spacing (mm)	Flip angle	spatial resolution	scan time
SSFP	3.6	1.8	260–325 mm^2^	192 × 193–232 × 230	4–6	− 3 ~ − 5	70°–80°	1.04 × 1.04 × 4	53 s
SSTSE	12,000	80	280–335 mm^2^	192 × 193–300 × 222	4–6	0–0.5	90°	0.79 × 0.79 × 2	30 s
SSFP cine	2.7	1.34	280–310 mm^2^		8	− 6	65°	2.00 × 2.00 × 8	1 m and 0.6 s
PC	7.9	4.8	300	232 × 230	8	N/A	12°	1.25 × 1.25 × 8	0.49 s

*SSFP* steady-state free-precession, *SSTSE* single-shot turbo spin echo, *PC* phase contrast, *TR* repetition time, *TE* echo time, *FOV* field of view.

**Figure 2:**
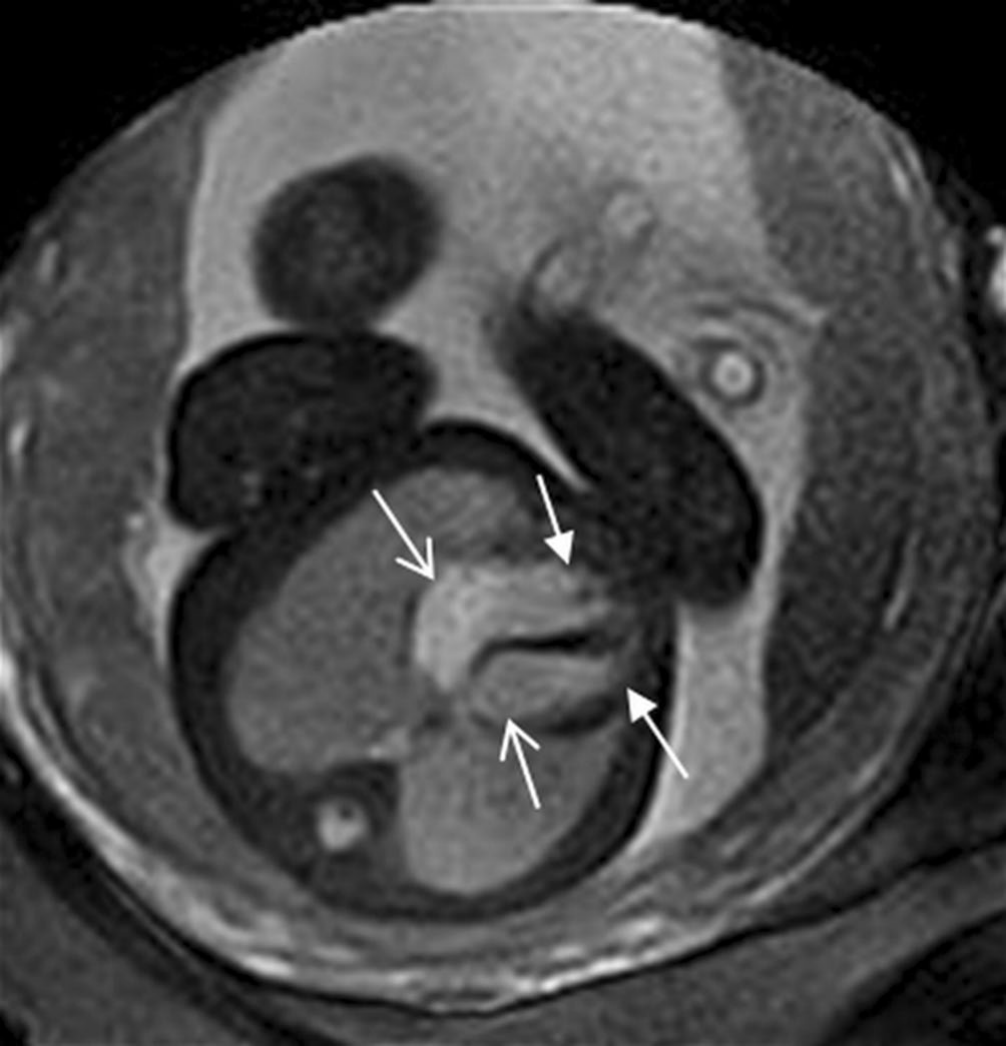
A 26-week-old foetus with a B-TFE sequence with radial k-space sampling. A foetal CMR B-TFE sequence with radial k-space sampling four-chamber view clearly showing a round FOV and the two ventricles (arrows) and two atriums (open arrows).

An SSFP sequence with overlapping slices was a key factor of the scanning technique. Overlapping slice scanning can improve the signal-to-noise ratio and the data can be used for reformation. The overlapping contiguous sections scan can be acquired directly in the Philips unit. In the GE unit, overlapping slice scanning was performed using frequency-selective fat saturation. Artefacts are common in foetal cardiac MRI. Sometimes, we combined the SSFP sequence with the radial k-space sampling technique to decrease the number of artefacts (Fig. 2).

No sedation, contrast media, or foetal cardiac gating was used in any of the cases in the present study. The mothers usually breathed freely and tolerate the examination in a supine position without any preparation. After a scout acquisition, a series of images (axial, sagittal, coronal, short-axis, and four-chamber views) were obtained for each foetus. The examination usually lasted about 30–40 min.

### Cardiac MRI reconstruction

We used a multi-planar reformatting reconstruction technique to reconstruct the foetal cardiovascular MR images. The transverse, coronal, or sagittal overlapping two-dimensional (2D) SSFP images can be used for the reconstruction. We reconstructed different imaging views, such as reconstructing a sagittal image from multiple transverse view images. If the imaging was insufficient for diagnosis, we performed reconstruction in the same direction according to the imaging with the original data and reconstruction angle less than 30°. The reconstructed images were clear enough to assess the foetal cardiovascular structures. We also used motion correction to correct any malposition caused by foetal movement and acquired images from different angles to evaluate foetal cardiovascular structures.

### Cardiac MRI evaluation

All foetal cardiovascular MR images were interpreted in consensus by two authors (S.Z.Dong and M. Zhu). S.Z. Dong had 18 years of experience in foetal US imaging and 16 years of experience in foetal cardiac MRI, M. Zhu had 42 years of experience in paediatric cardiac imaging and 16 years of experience in foetal cardiac MRI. Foetal cardiac structures were analysed using a modified anatomic segmental approach for CHD. The following components of the foetal cardiac anatomy were analysed^6^:

1. Visceroatrial situs, position of the heart, cardiac apex, and cardiac axis.
2. Position of the inferior vena cava, liver, stomach, bowel, and aorta relative to the midline; and the presence, appearance, and number of spleens.
3. Ventricular looping, ventriculo-arterial connections, and systemic and pulmonary venous connections.
4. Intracardiac structures.

By comparing the findings of foetal cardiac MRI with postnatal imaging or surgery, we calculated the diagnostic accuracy of foetal cardiac MRI.

## Results

**Figure 3:**
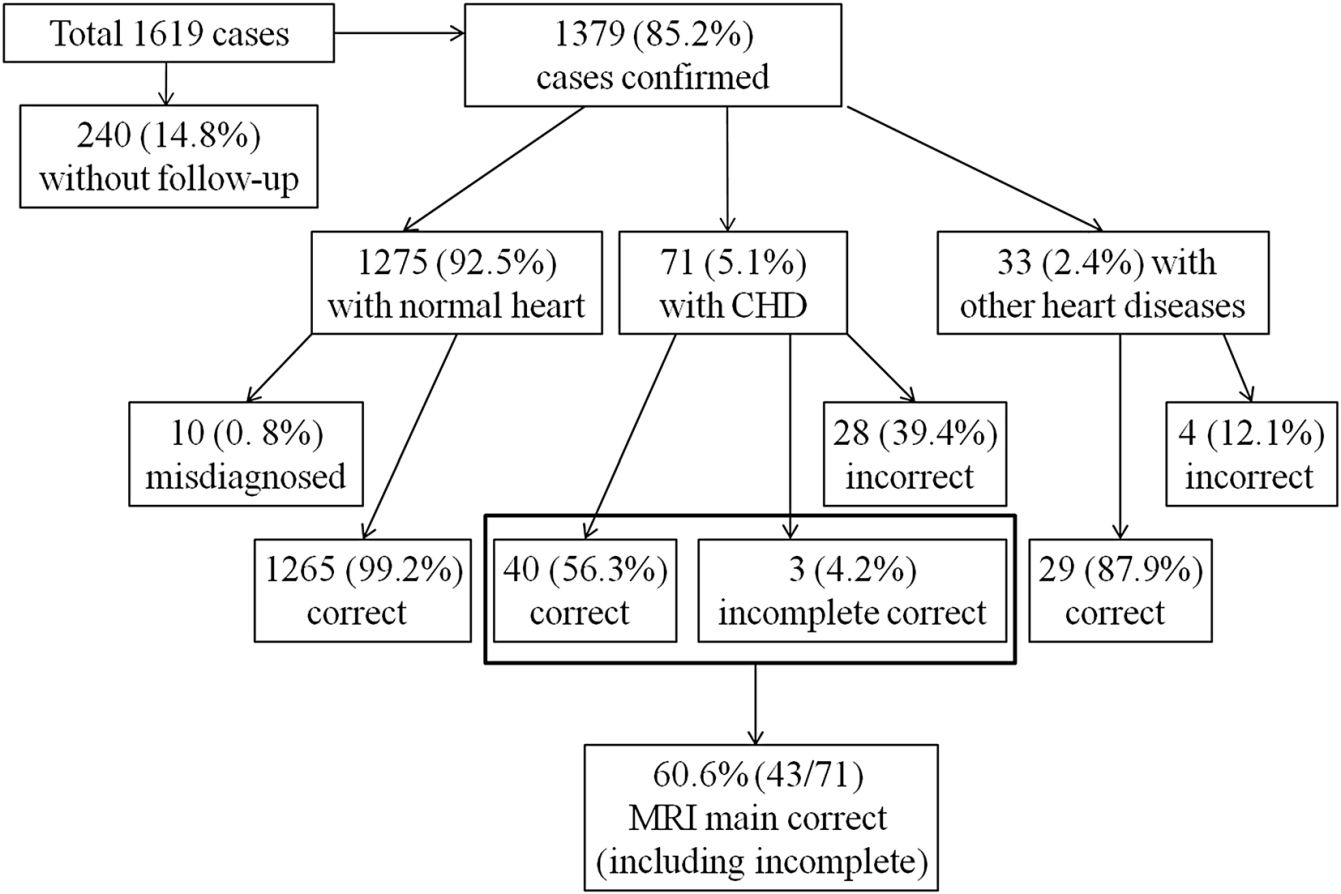
Diagram demonstrating all cases in this study. Diagram demonstrating all cases interpreted by foetal cardiac MRI and confirmed by postnatal diagnoses.

Among the 1,619 foetuses referred for cardiac MRI, 240 (14.8%), including three twins, had no follow-up confirmation because of pregnancy termination without autopsy or loss to follow-up. Thus, 1,379 (85.2%) cases were confirmed using postnatal imaging and/or surgery. Among the 1,379 cases, 1,275 (92.5%) cases were confirmed as normal at follow-up, 71 cases (5.1%) had CHD, and 33 cases (2.4%) had other heart diseases (Fig. 3).

Of the 1,275 cases confirmed to have a normal cardiovascular structure, foetal echo results have no definitive diagnosis for these cases. Foetal echocardiography findings were inadequate to show the four chambers of the heart and the outflow tracts of the two ventricles in 907 (71.1%) cases, only inadequate to show the four chambers of the heart in 102 (8.0%) cases and only inadequate to show the outflow tracts of the two ventricles in 266 (20.9%) cases with suspected normal four chambers of the heart. 1,265 (99.2%) of 1,275 cases were also interpreted as normal using cardiac MRI and 10 (0. 8%) of 1,275 cases were normal but interpreted as CHD by cardiac MRI. The 10 cases misdiagnosed by foetal cardiac MRI included 5 cases misdiagnosed as ventricular septal defect (VSD), 3 cases misdiagnosed as coarctation of aorta (CoA), and 2 cases diagnosed as pericardium effusion (postnatally normal) (Fig. 3).

**Figure 4:**
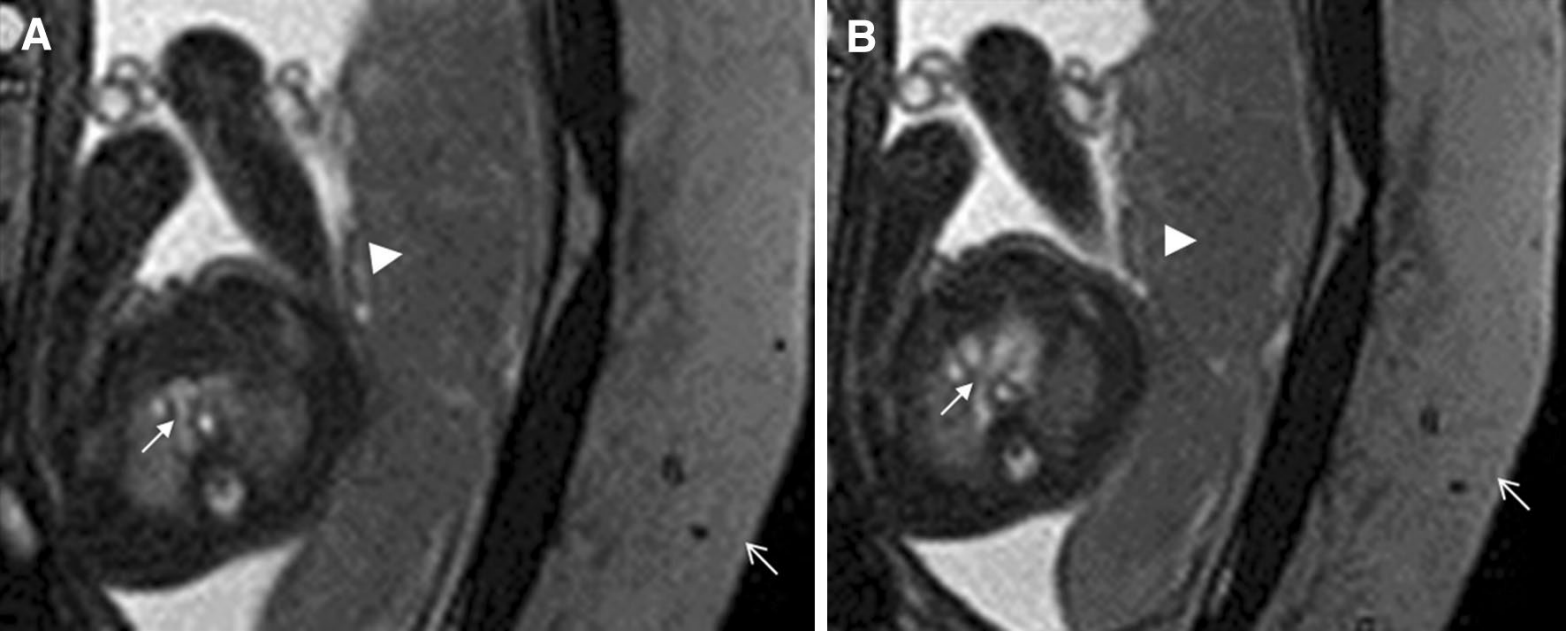
A 25-week-old foetus with a right aortic arch and right ductus arteriosus. Foetal CMR B-TFE transverse view of the aortic arch image showing the right-sided aortic arch (**A**, arrow) and the right-sided ductus (**B**, arrow), maternal obesity (**A**,**B**, open arrow), and placenta located in the anterior uterine wall (**A**,**B**, arrowhead).

**Figure 5:**
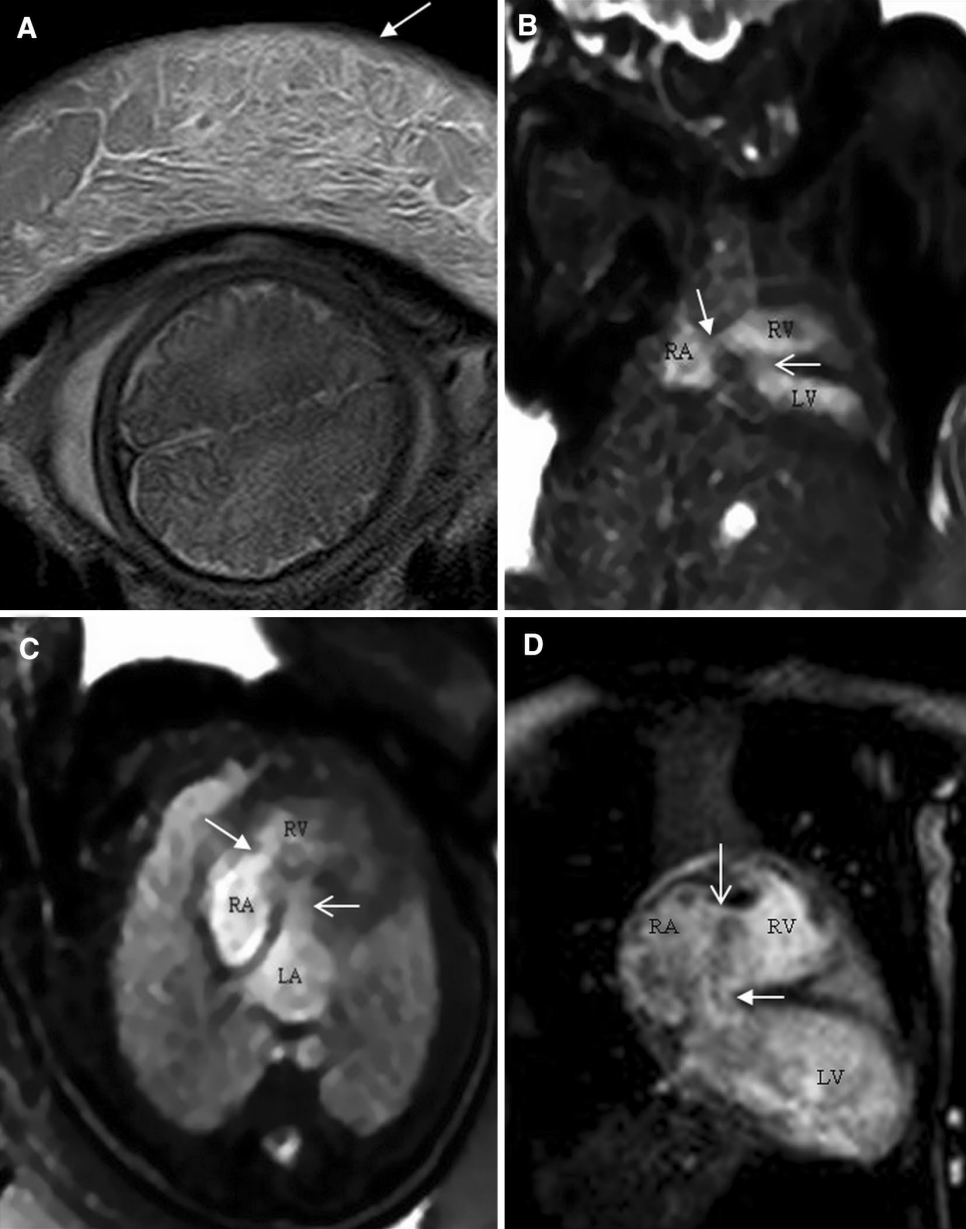
A 34-week-old foetus with a criss-cross heart. A 34-week-old foetus had a echocardiography at 22 weeks of gestation that suggested the presence of transposition of the great arteries (D-TGA) and ventricular septal defect (VSD). Follow up foetal echocardiography at 34 weeks of gestation was limited because of increasing maternal subcutaneous oedema and obesity. An axial SSTSE image of the maternal abdomen demonstrating oedema of the subcutaneous soft tissues (**A**, arrow). SSFP coronal and axial view images showing the right ventricle located superiorly (**B**, RV), the horizontal ventricular septum with a VSD (**B**, open arrow), the right atrium (**B**,**C** RA) connected to the right ventricle (**B**,**C** RV) in a right-to-left direction (**B**,**C** arrow), and the left atrium (LA) connected to the left ventricle (LV) in a posteroanterior direction (**C**, open arrow). A postnatal enhanced cardiac MR image showing a criss-cross heart (same as the prenatal cardiac MRI). The right ventricle was located superiorly (**D**, RV), the horizontal ventricular septum with a VSD (**D**, arrow), the right atrium (**D**, RA) was connected to the right ventricle (**D**, RV) in a right-to-left direction (**D**, open arrow). The left atrium (LA) was connected to the left ventricle (LV) in a posteroanterior direction.

**Figure 6:**
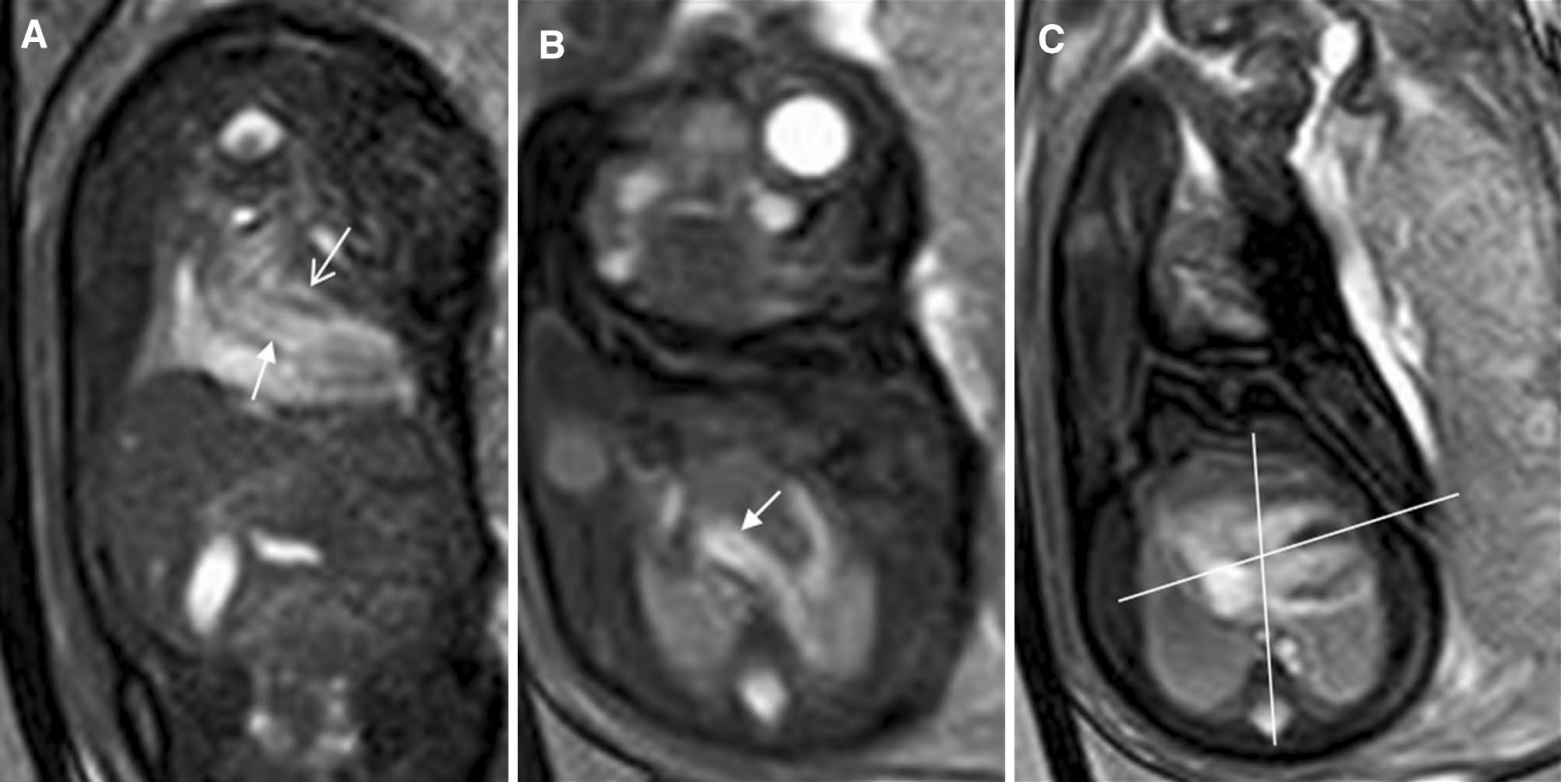
A 28-week-old foetus with a TOF referred for a foetal cardiac MRI because of oligohydramnios. Foetal CMR SSFP coronary and axial view images showing the oligohydramnios and stenosis of the right ventricle outflow tract (open arrow in **A**), the enlarged ascending aorta (arrow in **A**), the aortic arch (arrow in **B**), and the turning right cardiac axis (the angle between the line through the foetal spine and sternum and the interventricular septum in **C**).

Of 71 cases confirmed to have CHD, foetal echo results also have no definitive diagnosis for these cases; however, foetal echo results have diagnostic clues in 15.5% (11/71) cases (Table 3). Foetal cardiac MRI diagnoses were correct in 56.3% (40/71) of foetuses confirmed to have CHD, including three VSDs, three atrioventricular canal defects, one Ebstein malformation, five right aortic arches (Fig. 4), one double aortic arch, two CoAs, one interruption of the aortic arch, one pulmonary stenosis, one PA/VSD, five persistent left superior vena cava (PLSVC), two anomalous pulmonary venous connections, three Tetralogy of Fallot (TOF), four TGA, three double-outlet right ventricles (DORV), three HLHS, one asplenia syndrome, and one polysplenia syndrome (Table 3). In 4.2% (3/71), the main diagnoses were incomplete: one crisscross heart with missed unclosed ductus arteriosus (DA) at birth (Fig. 5), one TGA with missed CoA, and one TOF with missed unclosed DA at birth (Fig. 6). In 39.4% (28/71), the diagnoses by foetal cardiac MRI were incorrect (Fig. 3). The cases incorrectly diagnosed included six VSDs, five atrial septal defects (ASDs), six unclosed DA at birth , three CoAs, two pulmonary stenoses, one PA/VSD, three anomalous pulmonary venous connections, one anomalous origins of coronary arteries from the pulmonary artery, and one DORV (misdiagnosed as TGA) (Table 3). Fetal echo results have no definitive diagnosis for these cases, however, some cases still have diagnostic clue.

**Table 3:** Frequency of confirmed 71 fetal congenital heart diseases (CHDs) and comparison of the diagnoses between fetal heart magnetic resonance imaging (MRI) and follow-up.

Frequency of CHD	No. of fetuses	Fetal echo confirmed by follow-up	Fetal cardiac MRI diagnoses confirmed by follow-up
Ventricular septal defect	9	2 suspicious, *7 unclear*	*6 incorrect*, 3 correct
Atrial septal defect	5	*All unclear*	*All incorrect*
Atrioventricular canal defect	3	1 suspicious, *2 unclear*	Correct
Ebstein malformation	1	Suspicious enlarged right atrium	Correct
Unclosed ductus arteriosus	6	*All unclear*	*All incorrect*
Right aortic arch	5	*All unclear*	All correct
Double aortic arch	1	*Unclear*	Correct
Coarctation of the aorta	5	*All unclear*	*3 incorrect*, 2 correct
Interruption of the aortic arch	1	*Unclear*	Correct
Pulmonary stenosis	3	*All unclear*	*2 incorrect*, 1 correct
Pulmonary atresia with VSD	2	*Unclear*	*1 incorrect*, 1 correct
Persistent left superior vena cava	5	2 suspicious enlarged coronal sinus, *3 unclear*	All correct
Anomalous pulmonary venous connection	5	2 suspicious enlarged coronal sinus	*3 incorrect*, 2 correct
Anomalous origins of coronary arteries from the pulmonary artery	1	*Unclear*	*Incorrect*
Tetralogy of Fallot	4	*All unclear*	*1 incomplete correc*t in (unclosed DA at birth missed), 3 correct
Transposition of great arteries	5	*All unclear*	*1 incomplete* (CoA missed ), 4 correct
Double-outlet right ventricle	4	*All unclear*	*1 incorrect (*misdiagnosed as TGA), 3 correct
Hypoplastic left heart syndrome	3	1 suspicious small left heart, *2 unclear*	All correct
Asplenia syndrome	1	Suspicious small left ventricle	Correct
Polysplenia syndrome	1	*Unclear*	Correct
Crisscross heart	1	Four-chamber view unobtained in foetal transverse plane of chest	*Incomplete correct* (unclosed DA at birth missed)

Thirty-three cases (2.4%) had other heart diseases, such as pericardium effusion, cardiomegaly, intracardiac rhabdomyomas, and cardiac positional anomalies. In this group, foetal echo results also have no definitive diagnosis for these cases; however, foetal echo results have diagnostic clues in 9.1% (3/33) cases, e.g. 2 cases of suspicious enlarged heart in the cardiomegaly, 1 suspicious cardiac positional anomaly; foetal cardiac MRI diagnoses were correct in 87.9% (29/33) and incorrect in 12.1% (4/33) of cases (Fig. 3). The cases incorrectly diagnosed included one cardiac rhabdomyoma, one congenital cystadenomatoid malformation (CCAM), one pulmonary sequestration, and one pericardium effusion.

## Discussion

Foetal echocardiography has been the mainstay of prenatal assessment tools for foetal heart evaluation. However, there is an established and longstanding desire for a reliable adjunct to echocardiography, because certain foetal and maternal factors can have a deleterious effect on the quality of echocardiography images, especially oligohydramnios, unfavourable foetal position, maternal abdominal wall oedema, maternal obesity, maternal uterine myomas, or twins^13,18^. In the present study, all cases analysed had certain foetal and maternal factors that led to technically inadequate echocardiography. In addition, assessment of the foetal heart is typically done between 18 and 24 weeks of gestation^19^, because the reduction of the amniotic fluid volume, the intensification of rib calcification, and foetal position may impair the quality of foetal echocardiography imaging in later phases of gestation. Unlike echocardiography imaging, foetal MRI is not affected by maternal and foetal conditions such as obesity and oligohydramnios, which particularly impair sonographic visualization of the foetal heart^9,15,20^. MRI can image the foetus in any plane, providing a large field of view of the foetus with excellent soft tissue images^4^. Although MRI has proven to be a useful tool to examine the foetal central nervous system and infant heart disease, there have been relatively few reports of foetal cardiac abnormalities diagnosed using MRI. The visualization of the foetal heart using MRI is very complicated because of the small heart size, the high foetal heart rate, and the foetal body motion^5^. In this study, foetal body movements were the main limiting factor for foetal cardiac imaging. We believe that new techniques must be developed to improve foetal cardiac MRI diagnosis dramatically in the near future^10–13^. During the last 14 years, we mainly used our special scanning technique without advanced equipment and new software to obtain acceptable results.

In this study, the SSFP sequence was the main sequence used to study foetal cardiac structures, as reported previously^6,14,15,20^. Visualization of the cardiac chambers and the great vessels on SSFP images is better than those on SSTSE images. The very short acquisition time and parallel imaging technique with the SSFP sequence resulted in sufficient overall image quality without maternal or foetal sedation. Our scanning techniques were easy to perform and did not use special methods, such as foetal cardiac gating and maternal breath holding. The SSFP sequence with overlapping slices was a key factor of the scan technique in the present study. Overlapping slice scanning improved the signal to noise ratio and the reformation using the data was better than that without overlapping slice scanning. This technique also avoided the structures lost due to foetal motion. SSFP sequences with overlapping slices and radial k-space sampling technique were used to decrease the number of artefacts and improve the image quality. SSFP sequences were more useful to identify the morphology of foetal cardiovascular structures and function, thanks to the excellent contrast between ventricular cavities and myocardium. In our experience, SSFP sequences allowed the characterization blood and fat tissue as high signal intensity structures, improving blood-to-tissue contrast at the endocardial surface and fat-to-myocardium contrast, respectively, at the epicardial border with a more precise evaluation of the myocardium, ventricular, and atrial septal thickness^21^.

Cine-MR sequences combining the SSFP technique with real-time measurements resulted in quick and successive acquisition, without triggering or mother’s breath-hold. Cine-MR sequences were used to obtain a dynamic and functional image of the foetal heart and were better at showing the regurgitation and jet flow^21,22^. PC-cine sequences were used to obtain the blood directions and the velocity of the great vessels. In this study, PC sequences did not use cardiac gating, the single slice imaging of PC sequences did not show the blood velocity and volume, only showing the blood direction. However, the blood direction information was helpful for differential diagnosis, such as LSVC and TAPVC.

In this study, we also used a multi-planar reformatting reconstruction technique to acquire different angle imaging of foetal cardiovascular structures. We could correct the malposition imaging because of foetal movement and obtain imaging at different angles to diagnose CHD.

In the all study cases, the foetal echocardiography findings were inadequate to make any diagnosis or to describe the foetal heart because of technically limitations. In this situation, foetal cardiac MRI could be performed.

In this study, in the 71 confirmed abnormal foetuses with CHD, the correct diagnosis (60.6%, 43/71) is not high, but was still helpful. These MRI results were acceptable if compared with other large series of congenital heart disease detection data^1,23^. We should consider some types of congenital heart disease, such as unclosed ductus arteriosus at birth and secundum atrial septal defect, to be impossible to diagnose prenatally using imaging methods. The 28 cases of incorrect diagnoses by foetal cardiac MRI included five atrial septal defects (ASDs) and six unclosed DA at birth. If these 11 cases are not included, only 17 cases were misdiagnosed. We should also consider some types of congenital heart disease, such as coarctation of the aorta, the prenatal diagnosis of which is very unreliable using imaging methods. After birth, the ductus closes, which might cause local excessive constriction of the aorta. Such changes can lead to difficulties in diagnosis using prenatal imaging method. For some complex congenital heart diseases, such as PA/IVS or severe TOF, DORV, or TGA, we did not make an accurate diagnosis using MRI; however, there was not much difference in postnatal treatment outcomes. Some abnormalities are small in structure, such as anomalous pulmonary venous connections and anomalous origins of coronary arteries from the pulmonary artery. In these cases, the diagnosis is difficult using imaging methods, including echocardiography.

We believe that VSD and PS should be identified by echocardiography without the limitation of the acoustic windows. We reviewed the foetal cardiac MRI again and still could not make most the VSD and PS diagnoses. MRI usually misses small VSDs and mild PS; therefore, we do not think that MRI should be used instead of echo as a first-line foetal heart imaging tool. In our cases, several CHDs, such as transposition of the great arteries, pulmonary atresia with intact ventricle septum, and hypoplastic left heart syndrome should be treated by surgery at the neonatal stage, and foetal cardiac MRI diagnosis was usually correct in these cases. Thus, we considered that foetal cardiac MRI could be useful as an adjunct to technically inadequate foetal echocardiography.

Our study had several limitations. This was a retrospective study and as such is subject to limitations associated with its study design. The number of cases was not enough. In this study, we only analysed the potential utility of foetal cardiac magnetic resonance examination as an adjunct to technically inadequate foetal echocardiography.

## Conclusions

Foetal cardiac magnetic resonance imaging (MRI) could become a useful adjunct to foetal echocardiography when echocardiography technically inadequate for diagnosis. The overlapping slice scan and the retrospective same direction, multi-planar reformatting reconstruction technique are key points of the scanning technique.

## Data Availability

The datasets generated during and/or analysed during the current study are available from the corresponding author on reasonable request.
